# Age-period-cohort analysis of the incidence of multiple sclerosis over twenty years in Lorraine, France

**DOI:** 10.1038/s41598-022-04836-5

**Published:** 2022-01-19

**Authors:** Brigitte Gbaguidi, Francis Guillemin, Marc Soudant, Marc Debouverie, Guillaume Mathey, Jonathan Epstein

**Affiliations:** 1 Inserm, CIC-1433 Clinical Epidemiology, CHRU de Nancy, University of Lorraine, 9 Allée du Morvan, 54505 Vandoeuvre-les-Nancy, France; 2 Département of Neurology, CHRU de Nancy, 29 Avenue du Maréchal de Lattre de Tassigny, 54000 Nancy, France

**Keywords:** Diseases, Neurology

## Abstract

Multiple sclerosis (MS) is a neurodegenerative disease of the central nervous system. An increase in MS incidence over time is reported in several regions of the world. We aimed to describe the evolution of the annual MS incidence in the Lorraine region, France, from 1996 to 2015 and to analyze potential components of a possible change by a temporal effect of age at MS onset, MS onset period, and birth cohort, overall and for each sex. Cases were identified from ReLSEP, a population-based registry of MS cases living in Lorraine, northeastern France, with MS onset between 1996 and 2015. Age-period-cohort modeling was used to describe trends in MS incidence. Annual age- and sex-standardized incidences were relatively stable: 6.76/100 000 population (95%CI [5.76–7.91]) in 1996 and 6.78/100 000 (95%CI [5.72–7.97]) in 2015. The incidence ratio between women and men was 2.4. For all time periods, the peak incidence occurred between ages 25 and 35 years. Age-period–adjusted cohort and age-cohort–adjusted period analyses did not reveal a period or cohort effect. The incidence of MS remained stable over the study period in Lorraine, and we could not identify any particular effect of disease onset period or birth period on this evolution.

## Background

The epidemiology of multiple sclerosis (MS) has changed since the middle of the twentieth century regarding temporal, geographical and demographic alterations in the patterns of disease, mortality rates and causes of death^1^. According to the Atlas of MS (2020), this neurodegenerative disease of the central nervous system reached a worldwide prevalence of 2.8 million in 2020^2^. MS is the most common non-traumatic disabling disease with an irreversible character and affects young adults, in particular women^3–5^. A combination of environmental and genetic factors could be triggering factors^6,7^, and they affect the evolution of the risk of MS.

The global prevalence and incidence of MS is increasing in many countries^8–10^. MS represents a social burden in countries where its incidence is high^11–13^ linked to an early loss of productivity^14^, with life expectancy slightly reduced due to therapeutic progress^15^. In France, the average annual direct costs associated with MS were estimated at €12,296 in 2014, a global health cost of about €1.2 billion per year^16^.

The development of McDonald's diagnostic criteria in 2001^17^, revised in 2005^18^, 2010^18^ and 2017^19^ allowed for diagnosing MS earlier in the disease trajectory ^20^ and closer to symptom onset, which can affect morbidity indicators toward early diagnosis.

The distribution of MS in the France territory is heterogeneous, with a predominance in the northeast versus the southwest^21^. An increase in incidence has been reported since 1990 in some departments in Lorraine, in the northeast, the MS risk ranging from 3.7 to 7/100,000 during 1990–2000^22^. In 2004, this region had a higher standardized incidence than the French average, which was 7.5/100,000 (7.3–7.6) in the same year, as assessed with national health insurance data^23^.

Understanding the temporal dynamics of the epidemiology of MS is important for investigations of potential etiological factors and for planning future service provision. Three distinct temporal factors are usually studied as risk factors in a population: age, period, and cohort effects. The effects of these factors tend to have different depictions and underlying biological interpretations for a disease with an undefined etiology. An age-specific effect would suggest that age-associated events and/or exposures affect the MS risk. A calendar period effect would imply different patterns of MS case ascertainment due to a particular exposure during the period and/or changing diagnostic criteria, and a differential birth cohort effect would suggest that risk factor profiles or exposure vary from one generation to the next. Thus, we simultaneously consider age, period and birth cohort as the three covariates affecting the risk of MS, as suggested by Clayton and Schifflers^24^. However, these three factors are not mutually exclusive because characterization of any two implies knowledge of the third. They can be studied together and then deconstructed independently of each other to assess the respective effect of each.

This study aimed to describe the evolution of the annual incidence of MS in the Lorraine region over the 20-year period from 1996 to 2015 and to analyze the potential components of the change in incidence by a temporal effect of age at disease onset, period of disease onset and birth cohort, for each sex.

## Results

### Description of the study population

A total of 3,525 incident MS cases were identified between January 1, 1996 and December 31, 2015 in the Lorraine area. The mean ± SD age at MS onset was 34.3 ± 11.3 years (range 5–74). The overall female-to-male ratio was 2.4. The relapsing–remitting MS (RR-MS) form occurred in 90.3% of women and 80.5% of men.

Cases were unequally distributed between age groups regardless of period, with a higher number of cases in young adults (Table 1).

**Table 1:** Distribution of new multiple sclerosis (MS) cases in Lorraine by period of MS onset during 1996–2015 (n = 3525).

Period					
Age group, years	1996–2000	2001–2005	2006–2010	2011–2015	Total
05–09	0	0	2	6	8
10–14	7	9	4	8	28
15–19	58	58	62	55	233
20–24	106	123	134	126	489
25–29	170	135	139	164	608
30–34	143	161	111	120	535
35–39	143	120	128	115	506
40–44	104	115	92	91	402
45–49	87	88	80	79	334
50–54	40	70	54	49	213
55–59	16	23	32	31	102
⩾60	15	20	9	23	67
Total	889	922	847	867	3525

### Evolution of the annual incidence standardized by age and by sex

**Figure 1:**
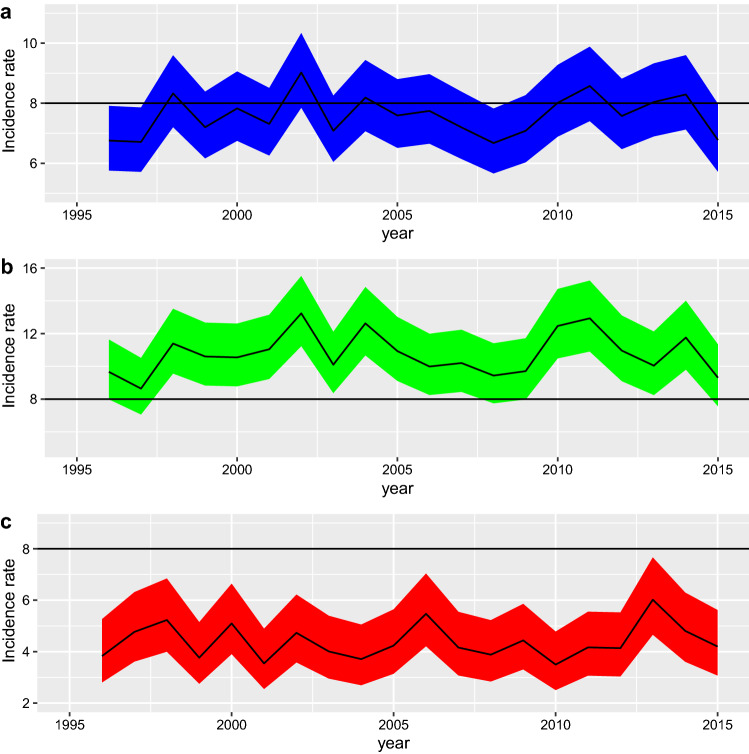
Change in overall annual sex- and age-standardized incidence of multiple sclerosis per 100,000 inhabitants in Lorraine from 1996 to 2015 (**a**), with 95% confidence intervals, and by sex standardized on age for women (**b**) and men (**c**), with 95% confidence intervals.

According to INSEE statistics, the population at risk of MS gradually declined, from 2,049,403 in 1996 to 1,992,170 in 2015 (i.e., a decrease of 2.65%). The female-to-male ratio ranged from 1.002 to 1.007. Over the 20 years, the overall trend in incidence of MS in Lorraine was relatively stable, with a difference in the distribution by sex (Fig. 1). The annual incidence rate per 100,000 inhabitants standardized for age and sex was 6.76 (95% CI [5.76–7.91]) in 1996 [women 9.66 (95% CI [7.99–11.63]) and men 3.83 (95% CI [2.81–5.27])] and 6.78 (95% CI [5.72–7.97]) in 2015 [women 9.31 (95% CI [7.57–11.34]) and men 4.20 (95% CI [3.07–5.62])]. It fluctuated between a minimum of 6.67/100,000 (95% CI [5.66–7.82]) in 2008 and a maximum of 9.02/100,000 (95% CI [7.85–10.34]) in 2002.

### Age-period-cohort modeling

#### Relation between age and period, cohort and period and age and cohort:

**Figure 2:**
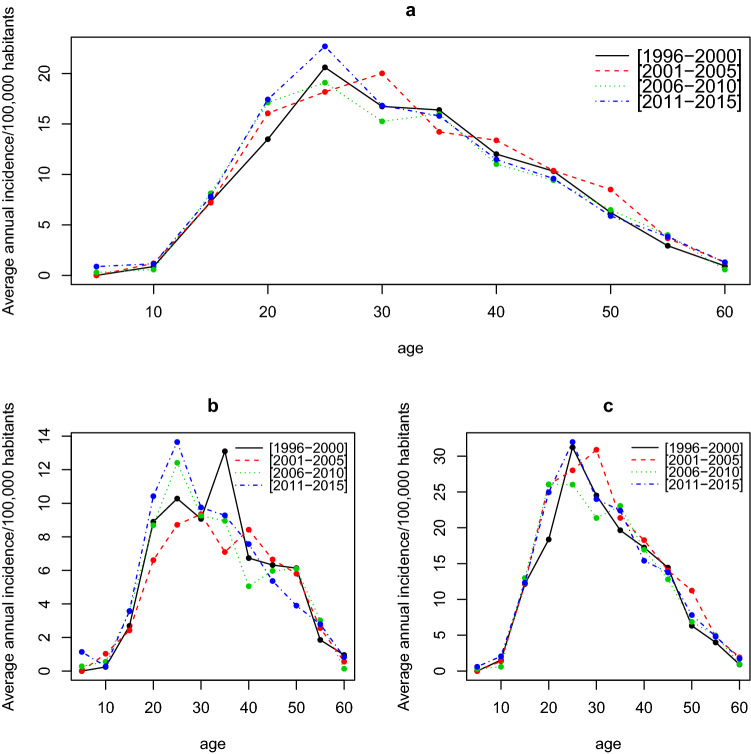
Time series of annual average incidence variation in multiple sclerosis per 100,000 population over age classes by period, all sexes combined (**a**), for men (**b**) and for women (**c**).

The distribution of the risk of MS in Lorraine was similar for all periods, as shown by the superposition of the age curves over the period brackets (Fig. 2a). There were peaks in the cumulative incidence rate at the key ages of the disease, between age 25 and 35 years (Fig. 2b,c).

**Figure 3:**
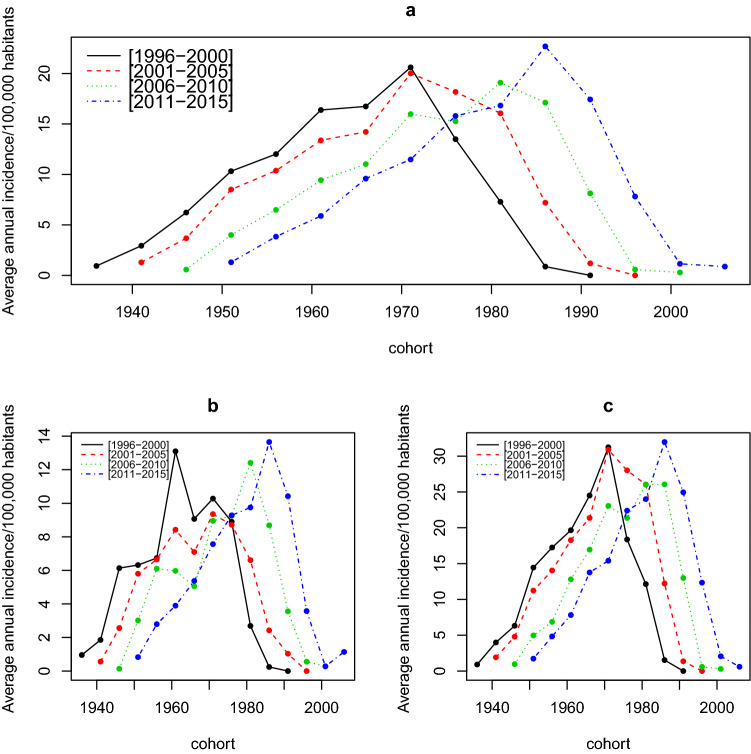
Time series of the change in annual average incidence for multiple sclerosis per 100,000 population during successive cohorts by period, all sexes combined (**a**), for men (**b**) and women (**c**).

Overall, the distribution by period according to cohorts did not show any variation (Fig. 3a). An analysis by sex found for men, for the period 2001–2005, a low incidence rate for the 1955 to 1970 cohorts compared to the other cohorts (Fig. 3b) as well as for women during 2006–2010 for the 1970–1985 cohorts (Fig. 3c). Rates for the 20- to 45-year age groups decreased during 2001–2005 (Fig. 3a) for both sexes and during 2006–2010 for women (Fig. 3c).

**Figure 4:**
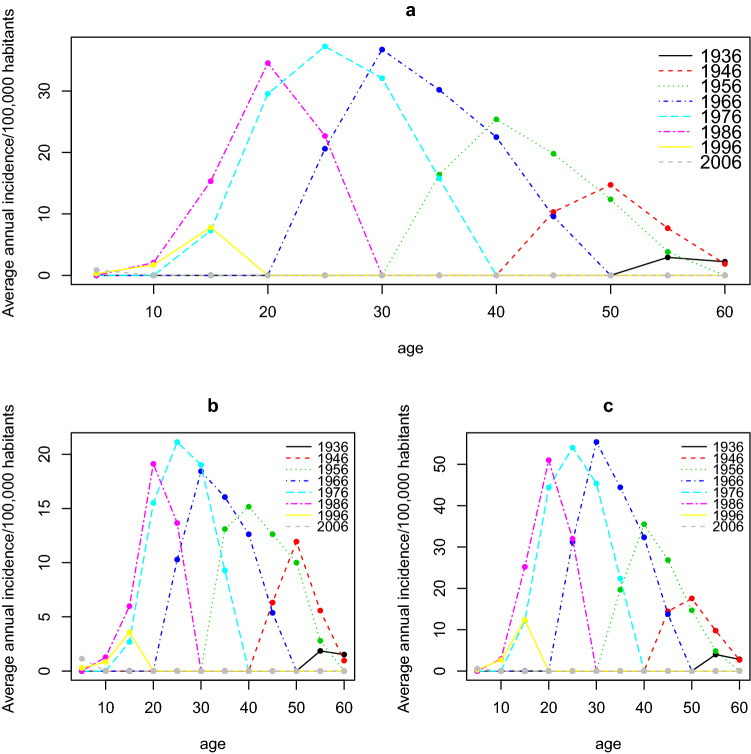
Time series of annual average incidence variation in multiple sclerosis per 100,000 population next age by birth cohort, all sexes combined (**a**), for men (**b**) and for women (**c**).

The age distribution of incidence rates had the same shape for the cohorts, with most people in the 20–40 age range during our study periods. For cohorts after 1996, our periods were too early to show peaks, and for cohorts before 1956, our periods were too late (Fig. 4).

#### Nested age-period-cohort analysis

Among the nested models (Table 2), the age-period-cohort model (*p* = 0.739), age-cohort model (*p* = 0.682), age-period model (*p* = 0.103), age-drift model (*p* = 0.098), and age alone model (*p* = 0.109) could not be rejected (*p* > 0.05), whereas the period-cohort model (*p* < 10^–3^) was rejected. Analysis of the specific model by sex (Table 3) had similar results. Neither the effect of the period of disease nor the effect of the birth cohort could be isolated, because none of the one-parameter models (Pd, Cd, P and C) fitted the data. We identified no trend by the different models.

**Table 2:** Deviance tables of age-period-cohort model all sexes combined.

Models	Deviance	*P* value	AIC
**APC**	15.632	0.739*	336.824
**AP**	43.554	0.103*	338.747
**AC**	18.403	0.682*	335.595
PC	958.563	< 10^–3^	1259.755
**Ad**	46.196	0.098*	337.389
Pd	2418.544	< 10^–3^	2693.736
Cd	960.403	< 10^–3^	1257.596
**A**	46.713	0.109*	335.905
P	2501.461	< 10^–3^	2774.654
C	960.797	< 10^–3^	1255.990
t	2421.055	< 10^–3^	2692.247
tA	2421.128	< 10^–3^	2690.320
tP	2503.951	< 10^–3^	2773.144
tC	2430.036	< 10^–3^	2699.229
1	2504.37	< 10^–3^	2771.562

*p ≥ 0.05.

*AIC* Akaike Information Criterion.

**Figure 5:**
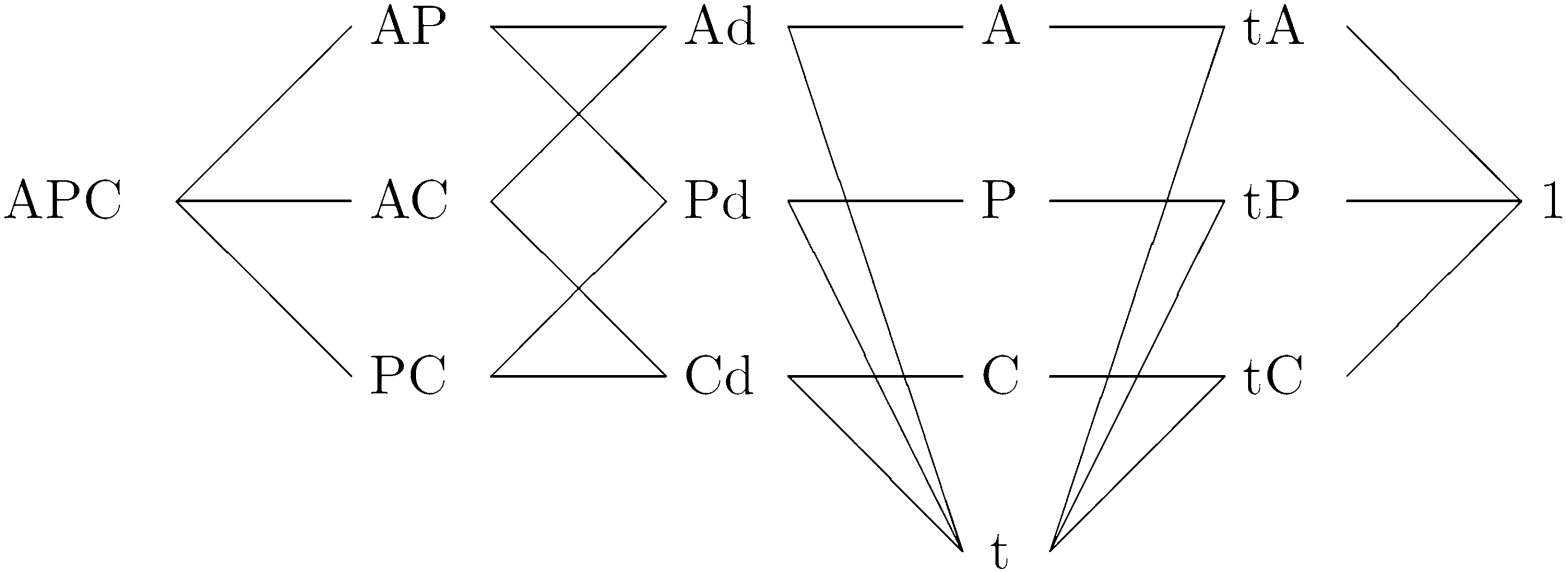
Diagram of the age-period-cohort model and its sub-models**.** The models are nested from right to left. *APC* age-period-cohort model, *AC* age-cohort model, *AP* age-period model, *Ad* age-drift model, *PC* period-cohort model, *Pd* period-drift model, *Cd* cohort-drift model, *A* age model, *P* period model, *C* cohort model, *A* age model, *t* trend model, *At* age-trend model, *Pt* period-trend model, *Ct* cohort trend model, *1*: intercept model. The term drift means that there must be some temporal variation in rates that cannot be interpreted as the effect of other non-specified parameters in the model.

All models are defined in the footnote of Fig. 5.

Analysis of the deviance of the age-period-cohort model for all sexes combined. The *p*-value of each nested model is analyzed. For *p* ≥ 0.05, the corresponding model cannot be rejected; the parameters contained in this model may have affected the evolution of MS incidence over the study period. Sub-models at *p* < 0.05 can be rejected: there is no effect of the parameters contained in this sub-model that is highlighted.

**Table 3:** Deviance table of sex-specific age-period-cohort model.

Models	Male deviance table			Female deviance table		
	Deviance	*p*	AIC	Deviance	*p*	AIC
**APC**	19.620	0.482*	285.076	13.315	0.863*	317.051
**AP**	40.861	0.163*	280.317	33.280	0.454*	311.015
**AC**	20.713	0.539*	282.169	19.475	0.616*	319.211
PC	324.017	< 10^–3^	569.473	672.247	< 10^–3^	955.982
**Ad**	43.635	0.150*	279.091	39.384	0.280*	313.120
Pd	701.807	< 10^–3^	921.263	1787.486	< 10^–3^	2045.221
Cd	326.121	< 10^–3^	567.578	677.155	< 10^–3^	956.890
**A**	43.842	0.173*	277.298	39.714	0.308*	311.450
P	706.954	< 10^–3^	924.411	1890.250	< 10^–3^	2145.985
C	326.127	< 10^–3^	565.583	678.127	< 10^–3^	955.862
t	704.058	< 10^–3^	919.514	1793.808	< 10^–3^	2047.543
tA	704.065	< 10^–3^	917.521	1793.864	< 10^–3^	2045.599
tP	709.208	< 10^–3^	922.664	1896.548	< 10^–3^	2148.284
tC	704.645	< 10^–3^	918.101	1804.434	< 10^–3^	2056.169
1	709.242	< 10^–3^	920.698	1896.951	< 10^–3^	2146.687

*p ≥ 0.05.

AIC, Akaike Information Criterion.

All models are defined in the footnote of Fig. 5.

Analysis of the deviance of the sex-specific age-period-cohort models. The *p*-value of each nested model is analyzed. For *p* ≥ 0.05, the corresponding model cannot be rejected; the parameters contained in this model may have affected the evolution of MS incidence over the study period. Sub-models at *p* < 0.05 can be rejected: there is no effect of the parameters contained in this sub-model that is highlighted.

On the basis of the AIC, the two best models were age and age-cohort (Table 2). Because as seen above, we found no cohort effect, the age-cohort model fit was probably a reflection of the age effect. Thus, the age-alone model was considered the best model, with neither a cohort nor period effect evidenced.

## Discussion

The present study was conducted on incidence data collected from the population-based ReLSEP registry in Lorraine, France, over a period of 20 years (1996–2015). The temporal analysis of the age- and sex-standardized annual incidence showed a relative stability in the annual rates of MS, with no clear upward or downward trend. The age-standardized sex-specific time analysis gave similar results.

In contrast to our study, other studies showed an increasing trend in incidence rate in some countries. In western Norway, the annual incidence increased from 1.9/100.000 (95% CI [1.2–2.6]) during 1953–1957 to 7.2/100.000 (95% CI [6.0–8.5]) during 1978–1982 and 8.5/100.000 (95% CI [7.3–9.7]) during 2003–2007^10^. Over 6 decades in Denmark, the incidence doubled in women, from 5.91/100,000 (95% CI [5.60–6.24]) to 12.33/100,000 (95% CI [11.91–12.75])^9^. However, in British Columbia, Canada, the incidence of MS was stable from 1996 to 2008, averaging 7.8/100,000 (95% CI [7.6–8.1]) per year^25^. British Columbia is among the regions with the highest incidence of MS in the world, as is the Lorraine region. Hence, the incidence of MS may not be sensitive to small changes in areas where the risk level is already high.

We found a predominance of RR-MS (87.4%), similar to most European studies^26^. The female-to-male ratio was 2.4 over the 20 years of our study. Overall the sex ratio was stable over the period, in contrast to other studies finding a marked increase in the female-to-male sex ratio of MS incidence in many countries over the past 50 years^4,27,28^. In Argentina, Rojas et al. found a variation of 1.8–2.7 over 50 years^4^, In Lithuania, Valadkeviciene et al. found a variation of 1.5–2^29^. This change in the female-to-male ratio is generally driven by an increasing MS incidence in women rather than a decline in that of men. Palacios et al. found a significant association between ratios of smoking prevalence for women and men in different countries and birth cohorts and corresponding ratios in MS incidence. The authors had estimated that smoking was associated with a 40% average increase in risk of MS^30^. Before World War II, few women smoked, but the number of women smoking rapidly increased post-war in industrialized countries^30^. This discrepancy between our study and others could be explained by our relatively limited period of observation, during which environmental exposures did not really change. This is the case for smoking rates for women, which were quite stable over the last decades in France^31^.

The APC analysis of the distinct contributions of age at MS onset, period of MS onset, and birth cohort retained only the effect of age without a period or cohort effect and no linear and non-linear trend over 20 years. This effect of age on the incidence rate was expected, as observed in this study and in the literature, with a peak of incidence between age 25 and 35 years.

Changes in lifestyle and environmental factors such as smoking^32–34^, vitamin D deficiency^35,36^ and childhood obesity (strongly correlated with vitamin D deficiency)^37–39^ over the birth cohorts represented in our dataset could lead to a change in the risk of one cohort category over another. In our birth cohorts, ranging from 1926 to 2010, no major changes were found over the entire study period. This finding suggests that the factors affecting disease risk have not changed significantly to affect disease risk trends in the Lorraine region or that there are mitigating factors not yet identified within the region. We also took into account the fact that the present study was based on a quasi-exhaustive registry and identified cases in the affected region in the year corresponding to MS onset. Most studies found an increase in incidence based on the year of diagnosis^8^ because the change in criteria improved the early identification of cases as well as the multiplication of sources of case registration^2^, or studies based on the date of MS onset^9,10^ were conducted over a wider period than ours. In contrast, this mode of case identification is more constant over time and guarantees a more accurate approach for detecting, or not, a trend in incidence over time.

The strengths of this study lie in our studying a large population during a relatively long period and using a quasi-exhaustive registry, the ReLSEP being the only MS registry recognized in France. Also, diagnoses were based on the newest criteria available at the time and made by neurologists. We used the year of MS onset as the year of incidence and not the year of diagnosis. We also considered a 5-year backward step to cover cases reported late in the registry because of the interval between the onset of symptoms and the reporting of the disease^40^.

One limitation might be that the observation period necessary to identify a change in incidence over time in a high-risk area was not reached. We did not have complementary data such as data on certain demographic factors, diet, and lifestyle (smoking) to concomitantly evaluate their evolution over time in the study population and their possible effect on the stability of incidence in the region. Future perspectives would be to maintain the operation of the registry while enriching it with data that could allow for etiological investigations if, in the long run, a modification of the incidence was detected.

In conclusion, this population-based study in Lorraine, a region classified at high risk for MS in France, did not find any trend in MS incidence during 1996 to 2015. This incidence was relatively stable in men and women, with a similar age effect on the distribution of risk within the general population. We did not show an influence of study period or a temporal variation potentially related to the birth cohort effect. Further study after a longer period of time, while linking the registry with data on factors that may affect the incidence of the disease, would bring further insight.

## Material and methods

This was an observational study based on the reporting of MS cases in Lorraine from January 1, 1996 to December 31, 2015 from a regional population-based quasi-exhaustive MS registry (ReLSEP: Registre Lorrain des Scléroses En Plaques).

### Study setting

Lorraine is an administrative region located in the northeast of France that had four departments during the study period. Lorraine had 2,311,655 inhabitants on January 1, 1999^41^. Since the end of the 1990s, the Lorraine region has been experiencing a dynamic population shift between a gain linked to a reduction in its migratory deficit, whereby it gained nearly 3400 inhabitants per year until 2011, and a slow decline linked to a low birth–death natural balance and a negative migration balance^42^.

### Data sources, completeness and regulatory aspects

Created in 1996 and covering northeastern France, the ReLSEP registry received its certification from the French registry authorities in 2009 and authorization (No. DR-2014–501) from the French National Commission for Data Protection and Liberties (CNIL). It contains longitudinal clinical and demographic follow-up data for MS patients in the region. Data were collected by using the standardized European Database of Multiple Sclerosis (EDMUS) software^43^. Cases were identified from hospitals, neurologists in the region, physical medicine and rehabilitation facilities, the national health insurance data, cerebrospinal fluid biochemical analysis laboratories, and the regional MS care network with a quasi-exhaustive representation^40^ after the multiplication of sources in 2014^44^. The onset of MS was medically confirmed according to the criteria of Poser et al. (1983) or McDonald et al. (2001) and modified by Polman et al. (2005, 2010). The procedure for identifying reported cases in Lorraine departments was extended to neighbouring departments. Each patient included in the ReLSEP was informed and asked to sign a written consent for reporting in the registry.

### Data management

Inclusion criteria were classification as an incident MS case between 1996 and 2015 and living in Lorraine during the considered period. The forms of initial diagnosis were RR-MS and primary-progressive MS (PP-MS). We excluded patients with neuromyelitis optica and radiologically and clinically isolated syndromes not reported as MS.

Data concerning all cases identified over the study period were extracted by using EDMUS software on January 1, 2021. The variables of interest were demographic data (age, sex), date of MS onset represented by the first symptom corresponding to the year of incidence, and the MS type at the time of diagnosis (RR-MS, PP-MS).

Data on the Lorraine population per year, by age groups of 5 years stratified by sex were extracted from the national census at the National Institute of Statistics and Economic Studies (INSEE)^45^.

### Statistical analysis

The MS cases and reference population data were classified into 12 age groups of 5 years each, from 5–9 to ≥ 60 years (i = 1, 2, 3, …I) and four periods of 5 years each from 1996–2000 to 2011–2015 (j = 1, 2,3, … J) based on their respective year of MS onset. This 5-year age and period classification was used to create K = 15 birth cohorts (K = I + J − 1)^46^. The corresponding population groups were constructed by averaging the population estimates on January 1 of the year under consideration and on January 1 of the following year to produce a relevant population denominator. The annual age- and sex-standardized incidence rates of MS were calculated from these data. The age-standardized sex-specific incidence rates were calculated by the direct standardization method^47^ with the French population structure on January 1, 1999, used as a standard. The confidence intervals for the standardized incidence rates were calculated with the Gamma distribution method^48^.

### Age-period-cohort modeling

Age-period-cohort models have been widely used in epidemiology for several decades. The models have three variables: age, period and cohort. The incidence rate modeling uses a generalized linear model with a predictor of the following form^49^:

$$ {\upmu }_{{{\text{i, k}}}}  = {\upalpha }_{{\text{i}}}  + {\upbeta }_{{\text{j}}}  + {\upgamma }_{{\text{k}}}  + {\updelta }. $$

The predictor μ_i,k_ is constructed from time effects for age, *α*_i_; period, β_j_; and cohort, γ_k_ and an error term, δ. However, these time effects cannot be fully recovered from the predictor because of the identification problem. In other words, knowledge of the predictor from the likelihood, which is a function of the predictor, is not enough to reveal the time effects. This identification problem can be circumvented by a parsimonious parametrization^50^. This parametrization allowed for the formulation of 14 nested sub-models within the age-period-cohort model (Fig. 5). The deviance of the sub-models is calculated against a chi-square distribution, its p-value and the Akaike Information Criteria (AIC) associated with the log-likelihood ratio statistics for the sub-models against the age-period-cohort model.

Analyses were conducted with R 3.4.2. APC analyses were conducted with the apc package 2.0.0. All methods were carried out in accordance with relevant guidelines and regulations.

### Ethics approval

The data are extracted from a population-based register certified by the French registry authorities in 2009 and approved by the French National Commission for Data Protection and Liberties (CNIL).

## Data Availability

All data are available from the authors on reasonable request.
